# ASO Author Reflections: Diagnostic Significance of Extramural Venous Invasion in Patients with Locally Advanced Esophageal Cancer

**DOI:** 10.1245/s10434-018-6985-5

**Published:** 2018-10-31

**Authors:** Zohra Faiz, Gursah Kats-Ugurlu, John T. M. Plukker

**Affiliations:** 1 Department of Surgery, University of Groningen, University Medical Center Groningen, Groningen, The Netherlands; 2 Department of Pathology, University of Groningen, University Medical Center Groningen, Groningen, The Netherlands

## Past

The presence of tumor cells in blood vessels, particularly extramural venous invasion (EMVI), is an independent poor prognostic factor in colorectal cancer (CRC).^1^,^2^ The Association of Directors of Anatomic and Surgical Pathology and the College of American Pathologists define EMVI as the microscopic presence of tumor cells in venous vessels beyond the muscularis propria.^3^, ^4^ The prevalence and significance of EMVI in esophageal cancer (EC) is still unclear. Most studies focused on differentiating venous invasion from lymphatic vascular invasion (LVI), as expressed in the current TNM classification, without assessing where the venous invasion was located. Problems addressed included the prevalence and prognostic significance of EMVI in EC resection specimens, and how to overcome difficulties among pathologists in identifying EMVI by using Elastica van Gieson (EVG) staining. We investigated archival specimens with pathological T3 or higher from patients operated by surgery alone, and those after neoadjuvant chemoradiotherapy (nCRT).^5^ The key question was whether EMVI can be used as a predictive factor in the response evaluation of nCRT.

## Present

EMVI was present in one-quarter of EC patients after surgery alone, and in 21.6% of patients after nCRT, as confirmed by additional EVG staining.^5^ The prevalence of EMVI was significantly higher in mid-esophageal and squamous cell carcinoma. Although significantly higher in the presence of LVI, EMVI showed no significant correlation with pathological T and N stage. In the nCRT and surgery-alone groups, EMVI was scored higher in tumors with lymphatic invasion (75% and 63%, respectively) and perineural invasion (both 75%). EMVI was shown to be a strong independent prognostic factor, with significantly shorter disease-free survival in the surgery-alone group with respect to EMVI-negative tumors. However, in the nCRT group, the presence of EMVI was not independently associated with survival. Based on these results, it seems necessary to differentiate EMVI from LVI in predicting prognosis. Therefore, pathologic reports should separately describe the presence of EMVI. Currently, EMVI can also be identified on diffuse-weighted (DWI) magnetic resonance imaging (MRI) in CRC patients.^6^,^7^

## Future

In future, EMVI should be investigated in a larger group of EC patients undergoing nCRT followed by surgery. Our results in the nCRT group are probably influenced by case mix and less power in a relatively small group with a potential selection bias of non-responders to nCRT.^5^ The value of EMVI as an independent predictor of response to nCRT remains questionable. To determine the impact of nCRT, we require accurate information about the presence of EMVI in the pre- and post-CRT setting. Therefore, we recommend investigation and correlation of EMVI in EC patients in ongoing or upcoming DWI–MRI studies. For a complete pathologic examination, separate reporting of EMVI in the EC resection specimens should be added. Moreover, as regression of EMVI after nCRT leads to vessel fibrosis and can be observed on MRI, it may be used as a predictive imaging marker in the response evaluation.
